# Deciphering the microenvironment of adult neurogenesis: a perspective from neurodegenerative diseases

**DOI:** 10.1186/s40035-026-00546-4

**Published:** 2026-03-24

**Authors:** Zheng-Kai Lao, Nan-Jie Xu

**Affiliations:** 1 Department of Clinical Laboratory, Songjiang Research Institute, Shanghai Key Laboratory of Emotions and Affective Disorders, Songjiang Hospital Affiliated to Shanghai Jiao Tong University School of Medicine, Shanghai, 201600 China; 2 Department of Anatomy and Physiology, Shanghai Jiao Tong University School of Medicine, Shanghai, 201318 China; 3 Key Laboratory of Cell Differentiation and Apoptosis of Chinese Ministry of Education, Shanghai Jiao Tong University School of Medicine, Shanghai, 200025 China

**Keywords:** Adult neurogenesis, Neural stem cells, Neurodegenerative diseases, Alzheimer’s disease

## Abstract

Neurodegenerative diseases are characterized by progressive neuron loss and brain atrophy. While conventional studies focused on neuronal death as the primary cause of these diseases, accumulating evidence suggests that impaired neurogenesis, particularly the dysfunction of adult neural stem cells (NSCs), may also contribute significantly to disease pathogenesis. Adult neurogenesis occurs primarily in two adult NSC niches. These specialized niches are enriched with complex cytokine networks, neuronal activity, and non-cellular components such as the extracellular matrix. Understanding the regulation of NSCs in the adult brain and how their dysregulation exacerbates neurodegeneration provides novel insights into therapeutic strategies. This review proposes that dysfunction of the NSC microenvironment, rather than neuronal death alone, may drive neurodegeneration, and that restoring this microenvironment offers a novel research direction of stem cell-based therapies.

## Introduction

Neurodegenerative diseases, such as Alzheimer’s disease (AD), Parkinson’s disease (PD) and Huntington’s disease (HD), are neuropathies with shared symptoms of neuron loss and brain atrophy. The long incubation period and low cure rate impose a great burden on the patients and their families. For a long time, studies of neurodegenerative diseases have concentrated on revealing the underlying mechanisms of neuronal death and searching for methods to restore pathogenic factors. Nevertheless, the failure of therapies that protect neurons from death forces us to reconsider other mechanisms underlying the irreversible neurodegeneration. Accumulating evidence supports the existence of functional neural stem cells (NSCs) in adult mammal brains [1, 2]. This offers a new possibility for neurodegeneration: apart from increased death of neurons, the reduced generation of newborn neurons may also contribute to the pathogenesis. The maintenance of adult neurogenesis is crucial for brain functions such as memory construction and affective control, while ablation or interference of adult neurogenesis leads to multiple cognitive defects [3–5]. Thus, disruption of adult neurogenesis is considered a potential pathogenic factor in various neurological disorders, especially neurodegenerative diseases. Although recent clinical studies have identified altered neurogenesis in patients with neurodegenerative diseases [6–8], how the NSC properties and numbers are changed remain largely elusive. Early clinical trials that directly transplanted NSCs or immature neurons to patients reported only limited efficacy in symptom improvement [9–12], implicating that NSC supply alone is insufficient to reverse neurodegeneration. The clinical failure underscores the urgency to understand how adult NSCs are regulated under physiological conditions and how these regulatory mechanisms become disrupted in distinct diseases.

Adult NSCs lie in the special microenvironment of the brain, known as NSC niches, that support and regulate the entire process of neurogenesis. In adult mammals, there are two acknowledged NSC niches, located in the subgranular zone (SGZ) within the dentate gyrus of the hippocampus and in the subventricular zone (SVZ) along the lateral walls of the lateral ventricles. The location of NSC niche determines the specific functions generated by adult neurogenesis. The microenvironment of NSC niches contains various regulatory factors including secreted or membrane-anchored cytokines, neuronal inputs, and the extracellular matrix (ECM), which modulate the behavior of NSCs and other intermediate cell types throughout the neurogenic process [13]. Disruptions of the microenvironment can significantly impair the regenerative capacity of NSCs, and as a result, contribute to cognitive symptoms of several neurodegenerative disorders.

In this review, we summarize the current knowledge of adult neurogenesis in both NSC niches, from structures to functions. We separate the regulatory factors of NSC niches into three major types: cytokine-based regulation, local neuronal activity, and non-biological signals from ECM. For each type, we first review disruptions of regulators associated with multiplex neurodegenerative diseases, and then discuss physiological mechanisms of how these factors modulate adult neurogenesis. Finally, we discuss the advances and drawbacks of recent trials of stem cell therapies, emphasizing the importance of reconstructing NSC microenvironment together with transplantation.

## Adult neurogenesis is a fundamental process regulated by niches

**Fig. 1:**
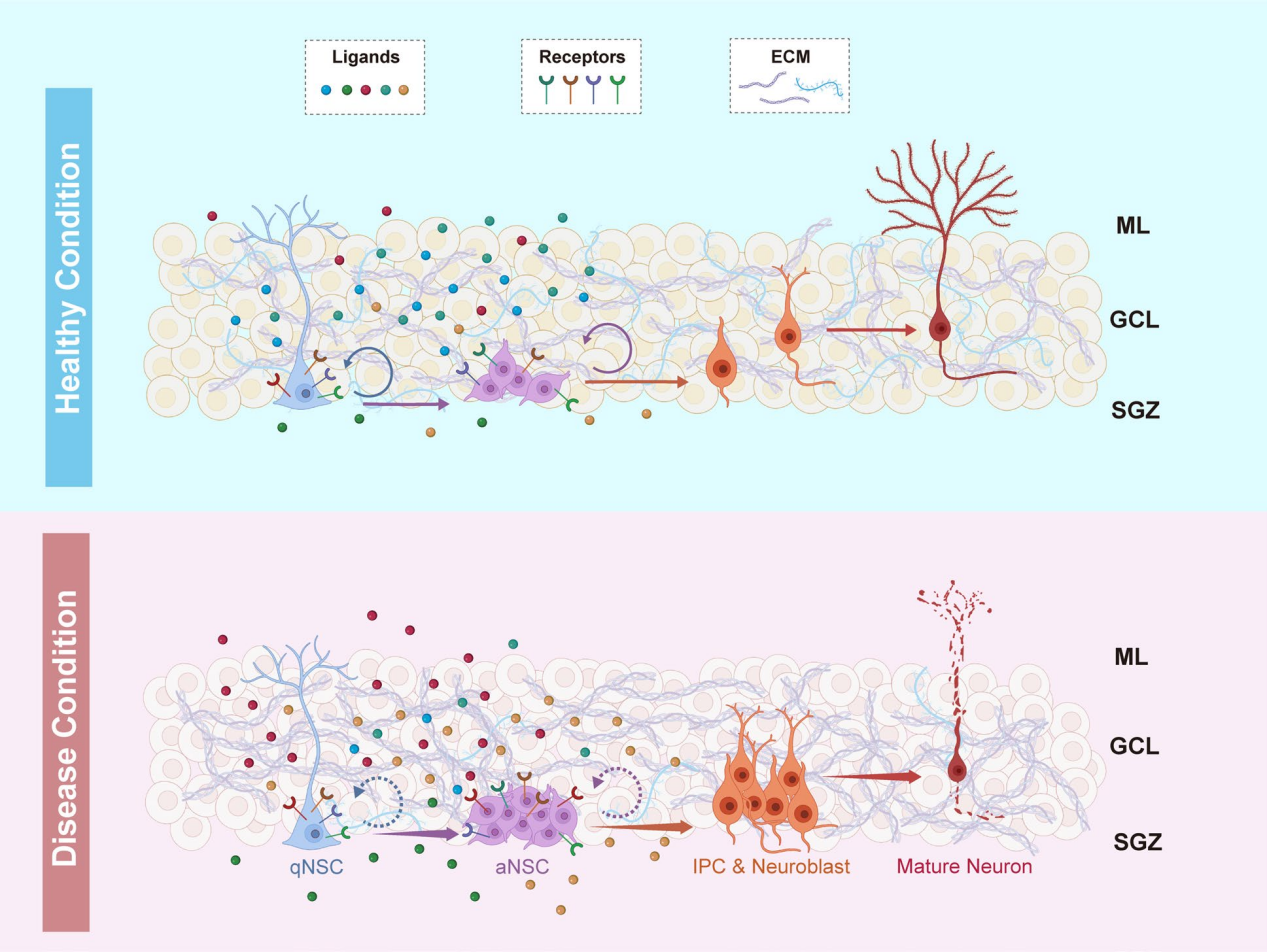
Diagram of SGZ neurogenic niche in healthy and disease conditions. In the upper panel of healthy brain, arrows indicate the direction of differentiation within complete neurogenesis, while round arrows imply the self-renewal of NSCs. Ligands indicate general signaling from niche cells to receptors on NSCs, where the colors represent the signal sources. ECM components locate surrounding niche cells. The lower panel represents a general disease condition. Enlarged arrows suggest premature differentiation. Dashed round arrows imply inhibition of self-renewal and maintenance of NSCs. The composition of extracellular signals is largely changed in the pathological environment. ML: molecular layer; GCL: granule cell layer; SGZ: subgranular zone

Adult NSCs are from a small population of NSCs that remain inactive from the middle period of embryonic development, named quiescent NSCs. The quiescent NSCs remain dormant in the developing brain but can become reactivated postnatally [14, 15]. Adult neurogenesis begins with activation of the quiescent NSCs settled in NSC niches. Once activated, the active NSCs proliferate for several rounds and then differentiate into progenitor cells of astrocytes, oligodendrocytes or neurons according to the regulatory signals they receive. The neural fate progenitor, named intermediate progenitor cells (IPCs), gives rise to neuroblasts. Neuroblasts then migrate to their target locations, where they mature as neurons and integrate into the existing neural network [16]. In such way, adult neurogenesis contributes to the ongoing neural plasticity and multiple cognitive functions of the adult brain (Fig. 1). Recent investigations of animal behaviors further elucidate the indispensability of sufficient neurogenesis for normal brain functions. Pharmacological inhibition of murine neurogenesis in the hippocampus interrupts memory formation of spatial tasks and is more sensitive to emotional disorders, such as anxiety and stress-related behaviors [17–19].

The regulation of adult neurogenesis relies largely on the NSC niche that protects and supports adult NSCs. Therefore, the composition of regulatory factors and the ultimate functions of neurogenesis vary across the two NSC niches, SVZ and SGZ. Located adjacent to the lateral ventricle of the brain, SVZ lies close to the cerebrospinal fluid (CSF) secreted by the choroid plexus, which is a special source of SVZ environmental regulators. CSF contains lipids, micro-RNAs, growth factors, and metabolites that regulate the behavior of NSCs within SVZ. On the other hand, SGZ is embedded in the dentate gyrus of hippocampus and surrounded by abundant neuronal network of hippocampus, where NSCs receive complex regulatory signals from active glial cells and neurons (Fig. 1). The high proximity to neurons enables direct neuronal regulation of neurogenesis in SGZ. As a result, newborn neuroblasts from SVZ/SGZ will migrate to olfactory bulbs or the hippocampal neuronal network, respectively, to support distinct brain functions [20, 21]. Since the hippocampus is the core of working memory formation and connects to multiple cortical regions controlling cognitive functions of the brain [22, 23], impairment of SGZ neurogenesis is more related to dementia-like symptoms compared to SVZ neurogenesis impairment. To summarize, the NSC niche provides a unique microenvironment to finely regulate the activity of NSCs, leading to the supply of sufficient and proper cells for the adult brain throughout the lifespan.

## Disrupted extracellular regulators in neurodegenerative diseases

In the NSC microenvironment, diverse extracellular regulators are the most effective and integral components that regulate the proliferation, differentiation, or survival of NSCs through ligand-receptor interactions. These regulators originate from astrocytes, oligodendrocytes, microglia, vascular tissue cells, or even the NSCs themselves. Disruptions of these cell-derived regulators are prevalent in neurodegenerative diseases, suggesting NSC dysregulation may be a common mechanism of irreversible neurodegeneration (Fig. 1).

### Neurogenic locus notch homolog protein (Notch) signaling balances quiescent and active NSCs

Membrane-bound Notch signals are well-studied regulatory factors for proliferation, cell fate determination and differentiation of pluripotent cells. Alterations in Notch signaling have been repeatedly observed across multiple neurodegenerative diseases.

In AD, both immunolabeling and quantitative analyses have revealed significant declines of the Notch1 ligand Jagged1 in the cortex, hippocampus, CSF and plasma of patients, which are highly correlated with cognitive impairment [24, 25]. In contrast, Notch1 signaling, particularly that expressed by reactive astrocytes, is upregulated in the spinal cords of amyotrophic lateral sclerosis (ALS) models and patients, while blockage of astrocyte-derived Jagged1 is sufficient to restore ALS symptoms in rodent models [26]. Notably, although the presence and traits of reactive astrogliosis in non-motor brain regions of ALS patients remain elusive, whether the reactive astrocytes appear in NSC locating regions needs to be further studied. As for PD, Notch1 transcription is repressed by α-synuclein accumulation in the hippocampus via p53 activation and direct promoter binding, whereas Notch2 expression is increased in patients’ serum [27, 28]. Furthermore, the Notch2 enhancer Notch homolog 2 N-terminal-like protein C (NOTCH2NLC) is reported as a potential biomarker of PD and neuronal intranuclear inclusion disease (NIID) [29, 30]. Although no study has yet directly examined NSC proliferation or neurogenesis in the adult SVZ or SGZ of NIID patients, the significant changes of neurogenic-associated NOTCH2NLC suggest the potential contribution of neurogenesis in the disease. Mutations in Notch3 represent another pathogenic mechanism, particularly in cerebral autosomal-dominant arteriopathy with subcortical infarcts and leukoencephalopathy (CADASIL). Notch3 point mutations are associated with white matter loss and dementia in patients and model mice [31, 32]. These findings highlight the context-dependent modulation of Notch signaling in distinct neuropathological environments.

There are four Notch receptors, known as Notch1, Notch2, Notch3 and Notch4, located on the membrane of NSCs. Under physiological conditions, Notch receptors are membrane-bound transducers that preserve the balance between quiescent and active NSCs within both SVZ and SGZ. Notch1 ligands such as Delta and Jagged are widely expressed on neighboring astrocytes, progenitors, and vascular cells, enabling local juxtracrine communication. Upon ligand binding, γ-secretase-mediated cleavage releases the intracellular domain of Notch1, which translocates into the nucleus to activate Hes1/3/5 transcription, thereby repressing genes for development and maintaining NSC quiescence [33, 34]. NSC-specific impairment of Notch1 receptors induces acute NSC activation and IPC proliferation in the adult brain, which rapidly consumes the adult NSC pool and results in the exhaustion of neurogenic activity within 3 months [35, 36]. Similarly, Notch2 activation maintains NSC dormancy through the Id4–Hes5 inhibitory loop, mirroring the suppressive effects of Notch1 on cell-cycle entry [37, 38]. Therefore, the overall reduction of Notch1/2 activities—normally serving as brakes on neurogenesis—may explain the early transient neurogenic hyperactivity but late deficient neurogenesis observed in PD and AD [39]. Notch3 signaling primarily maintains NSC quiescence by stabilizing stemness and ensuring asymmetric divisions of quiescent NSCs [32–34]. CADASIL-associated Notch3 mutations thus weaken this suppressive function, producing aberrant activation of NSCs and eventual depletion of the stem-cell reservoir [40–42]. In conclusion, the fine-tuned Notch cascade acts as a safeguard against premature exhaustion of adult neurogenesis, a mechanism relevant to multiple neurodegenerative conditions.

### Bone morphogenetic proteins (BMPs) and Wnt, a pair of secreted brake and accelerator signals

BMPs and Wnts are conventional diffusing growth factors present in multiple stem cell niches. Wnt signals activate developmental processes via intranuclear β-catenin translocation, while BMP receptors canonically phosphorylate SMAD (named from the protein family encoded by homologous *sma* gene of *C. elegance* and *Mad* gene of *Drosophila*) protein pairs to inhibit the translation of development-associated genes. Aberrant activity of BMP and Wnt signaling is a recurrent feature in neurodegenerative diseases, where these two antagonistic pathways shift in opposite directions to disturb the NSC homeostasis.

A clinical proteomic study analyzing brain samples from AD patients revealed increased levels of WNT5A and WNT5B, two canonical Wnt ligands, along with elevated levels of BMP negative regulators such as BMP3 and SMOC1 (SPARC-related modular calcium-binding protein 1). These aberrant signaling patterns emerge early in AD progression and accumulate as the disease advances. Although this study did not specify the affected brain regions, it highlights a dysregulated cytokine microenvironment characterized by BMP downregulation and Wnt upregulation in AD brains [43]. Impaired adult neurogenesis caused by insufficient Wnt signaling is also observed in other neurodegenerative diseases, including PD and HD, while Wnt deficiency-related demyelination in ALS may similarly reflect disruption of NSC function [44]. On the other hand, some data also suggest that BMP signaling protects dopaminergic synapses, raising the possibility of BMP-activating therapies for PD, although the therapeutic efficacy remains uncertain [45]. Pharmacological activation of the canonical Wnt downstream effector β-catenin effectively stimulates neurogenic activity and partially rescues disease symptoms of PD [46]. Further studies in *Drosophila* and rodents indicate that BMP signaling is elevated in the nerve terminals of HD and in reactive astrocytes of ALS [47, 48].

How do BMP and Wnt signals regulate NSC activity under physiological conditions? Within the NSC niche, Wnt ligands are secreted predominantly by NSCs and astrocytes, while NSCs themselves express multiple Wnt receptors. Overexpression of Wnt3 in rodents promotes NSC proliferation within the SGZ, thereby enhancing neurogenesis [49]. Physiologically, mature granule neurons in the SGZ express secreted frizzled-related protein 3 (sFRP3), a Wnt inhibitor, to maintain NSC quiescence. When granule neurons become active, sFRP3 expression decreases, facilitating NSC activation in response to increased neuronal requirements [50]. BMP ligands are produced by several niche cell types, including NSCs, intermediate progenitor cells, and surrounding support cells. In the adult NSC niche, BMP signaling functions as a classical brake maintaining NSC quiescence. Injection of the endogenous BMP antagonist Noggin or deletion of the effector SMAD4 leads to extensive NSC activation and proliferation, leading to rapid depletion of the stem cell pool and a decline of long-term granule-cell generation [51, 52]. Mechanistically, BMPs—particularly BMP4—promote degradation of the NSC activation marker ASCL1 (Achaete-scute homolog 1), thereby preventing premature NSC activation [53].

Overall, Wnt and BMP pathways act as an accelerator–brake pair that finely tunes neurogenic activity. Based on reports of Wnt/BMP alterations in disease, NSC activation appears to be enhanced in AD, while the Wnt/BMP balance is switched toward suppressing neurogenesis in PD, HD, and ALS, consistent with impaired nervous functions but not with the clinical observations of increased neural precursors [39]. Possible explanations include compensatory regulation of hyperactivity or stage-dependent discrepancies between clinical findings and mechanistic studies.

### Epidermal growth factors (EGFs) support the essential proliferation of NSCs

EGFs are a large family of diffusing growth factors in NSC niche, secreted by astrocytes, microglia, neurons, ependymal cells and endothelial cells. EGF receptors (EGFRs) are widely expressed in NSCs and neurogenic intermediate cell types, which receive multiple EGF inputs from the microenvironment and promote NSC proliferation. EGF dysregulation has been widely reported in neurodegenerative diseases. EGFRs are predominantly involved in AD pathogenesis. Aβ binding to EGFR leads to neurotoxicity or activation of neuroinflammatory responses in glial cells, thereby contributing to neurodegenerative pathology [54, 55]. Familial AD model mice exhibit a significant decrease of plasma EGF levels at the age of 8 months; peripheral replenishment of EGF restores the cerebrovascular structure and attenuates the progression of cognitive impairment while brain EGF levels are not changed [56]. As a main symptom of PD, dopamine depletion is reported to impair neurogenic activity via reducing EGF level in the SVZ of both PD models and clinical patients; cells expressing EGFR are significantly reduced in the SVZ of human PD patients compared with age-matched controls [57]. Excessive EGF administration shows neuroprotective effects in motoneuron-associated diseases, such as HD and ALS, which help rebuild the locomotor circuit in model mice to some extent [58, 59].

EGF signaling is clearly a strong basic enhancer of neurogenesis. Seven-hour in vitro infusion of EGF promotes the proliferation of active NSCs while terminating the differentiation of NSCs to neuroblasts [60]. Endothelia-derived betacellulin, another member of the EGF family, maintains the proliferation and expansion of IPC population, whilst blocking betacellulin significantly reduces the number of IPCs and neuroblasts [61]. Transforming growth factor-α (TGFα) can also bind to EGFR and promotes the proliferation of NSCs. Persistent TGFα administration converts a part of mature astrocytes into NSCs, though solid in vivo evidence is lacking [62, 63]. Given the extensive decline of EGF signals in multiple diseases, EGF appears to be a protective pathway of healthy neurogenesis. Several studies claim that extra EGF supplement could be a promising therapy to activate the silent neurogenic system in patients suffering from late-staged neurodegenerative diseases [56–59, 64]. Nevertheless, given the potent proliferative effects of the EGF signaling pathway and its regulation of various stem cell proliferation processes, monotherapy with EGF supplement carries a significant risk of oncogenesis. Therefore, EGF or EGFR agonists should be administered as an auxiliary agent in conjunction with other NSC-based therapies.

### Other diffusing extracellular signals spread in NSC niche

In addition to the conventional growth factors known to generally regulate stem cell lines, there are other secreted regulators spreading in the central nervous system that might contribute to the fine regulation of NSC quiescence and the neurogenic process. For instance, neuron-secreted brain-derived neurotrophic factor (BDNF) and insulin-like growth factor 1 (IGF-1) are essential for the fate determination of proliferating NSCs into neurons or oligodendrocytes, which further support the subsequent differentiation process [65, 66]. BDNF signals greatly decline in multiple neurodegenerative diseases, in particular AD, PD and HD, due to the drastic neuronal loss [67–69]. Such decrease occurs early before neurodegeneration and pathology like protein aggregation, suggesting that NSC dysregulation starts long before clinical symptoms. Attempts to genetically or pharmacologically inducing neurogenesis in AD models fail to rescue cognitive impairment unless BDNF is supplied to the neurogenic environment [70]. Pleiotrophin (PTN) was originally regarded as an oncogenic protein in the field of cancer research, especially glioblastoma. PTN in the brain can be secreted by quiescent NSCs and pericytes surrounding vessels [71]. Recent studies revealed that PTN also mediates neuroprotection. PTN breaks the cell cycle arrest of NSCs, accelerates progenitors to proliferate and promotes dendritic development of newborn neurons. PTN overexpression increases neuronal survival and ameliorates cognitive function. However, the mechanisms underlying PTN alterations in neurodegenerative diseases and the subsequent effects remain elusive [72, 73]. Notably, pericyte loss is extensively observed in AD, PD, HD, ALS and human immunodeficiency virus-induced neurocognitive disorders, implicating a potential deficiency of environmental PTN signals for NSCs [71]. Interestingly, a recent study reported contrast effects of PTN, where *Ptn* overexpression induced NSC dormancy and blocked normal neurogenesis in a bipolar disorder model [74]. More studies are needed to explore the extent of PTN alterations in neurodegenerative diseases and how adult neurogenic activities are affected.

### Microglia-associated inflammatory factors modulate NSCs

In addition to astrocytes and fibroblasts that modulate neurogenesis through cytokines, microglia are rising as a critical modulator of adult neurogenesis. Pharmacological ablation (tamoxifen and diphtheria toxin injection to Cx3cr1^CreERT2^/iDTR^f/f^ mice) or functional knockout (tamoxifen injection to Cx3cr1^CreERT2^/Casp3^f/f^ mice) of microglia in rodent brains sufficiently reduces the production of neuroblasts as well as newborn neurons, which further impairs the formation of short-term memory [75, 76]. Notably, the exact regulatory effects of microglia largely depend on their cell states. Physiologically, resting microglia reside within both NSC niches to phagocytose apoptotic newborn neurons without activating immune responses in the brain. Acute immune response or aging does not change the phagocytosis-associated neurogenesis [77]. Removal of such phagocytotic microglia transiently promotes the proliferation of neuroblasts but significantly reduces the activity of neurogenesis in longer time [78]. In addition to phagocytosis, resting microglia have the potential to regulate NSCs through secretion of multiple cytokines, chemokines and short peptides [78, 79]. In the presence of immune stimuli like LPS treatment, resting microglia transform into classically activated microglia, which can also engulf apoptotic neural progenitors and express a variety of proinflammatory factors including interleukin (IL)-1β, IL-6 and tumor necrosis factor α (TNF-α). The microglia-secreted proinflammatory signals would further induce neuronal apoptosis and attenuate the proliferation of NSCs, resulting in inhibition of neurogenic activity [80–82]. Interestingly, during the regenerative process, another activated type of microglia is generated, the alternatively activated microglia, which secret anti-inflammatory cytokines including IL-10, TGF-β and IGF-1 during tissue repairment [80, 81]. In contrast to proinflammatory factors, the anti-inflammatory cytokine treatment can induce NSC activation and de novo neuron generation [83–85]. Although acute microglial response is essential for brain regeneration after injury, chronic neuroinflammation leads to transition of more microglia to the classic activation than to the alternative activation state. Therefore, long-term neuroinflammation is much more detrimental for NSC maintenance and neurogenesis (Fig. 1).

Alterations of microglial activity have been extensively reported in neurodegenerative diseases. For instance, microglial overactivation has been detected in multiple brain regions of postmortem AD brains [86–88]. A large proportion of microglia are so stimulated that they lost their homeostatic marker Iba1. Translocator protein positron emission tomography (TSPO-PET) imaging in AD patients illustrated microglial overactivation even at early stages before symptom onset [89, 90]. At the same time, higher levels of IL-1β, IL-6, IL-18, TGF-β and TNF-α have been detected in serum from AD patients [91]. TSPO-PET imaging in PD patients also showed increased microglial activation and immune responses in the nigro-striatal pathway [92]. Postmortem analysis of PD brain samples, especially in the pathogenic striatal region, demonstrated elevated proinflammatory factors such as IL-1β, IL-2, IL-4, IL-6, TGF-α and TNF-α. Mechanistically, α-synuclein can stimulate microglia to secrete such cytokines [93]. Nevertheless, virus-induced microglial expression of anti-inflammatory *Il10* showed neuroprotective effects in a PD model [94]. In HD, reactive microglia are significantly increased in HD human samples and mouse models, especially in the stratum and cortex [95, 96]. Increased proinflammatory cytokines like IL-1β, IL-6 and TNF-α are widely reported in HD patients, whereas the elevation of the anti-inflammatory factor TGF-β is also detected, which might be a result of compensatory regulation for homeostasis [97, 98]. Furthermore, living imaging of patients with ALS or multiple sclerosis (MS) illustrated strengthened microglial responses, accompanied by significant increases of proinflammatory signals [99–102].

Taken together, microglia activity is increased in most neurodegenerative diseases, generating a chronic neuroinflammatory environment with proinflammatory signals. Since the proinflammatory environment inhibits neurogenesis, the expansion of classically activated microglia may inhibit NSC activities in neurodegenerative diseases, though straight evidence is missing at present.

## Abnormal neuronal regulation of NSCs in neurodegenerative diseases

NSCs are not only regulated by diffusive cytokines but also by neural activity signals. Compared to cytokines that can be synthesized by multiple cell types and take long-term effects, signals from neuronal activities, particularly neurotransmitters and neuromodulators from activated neurons, act as real-time local regulators within the NSC microenvironment. Recent studies have demonstrated diverse receptors on NSC membranes that can detect neuronal signals like glutamate, gamma aminobutyric acid (GABA), dopamine, serotonin and acetylcholine, mediating the activation of adult NSCs or the precursor maturation [103]. Given the proximity between NSCs and adjacent neurons, rapid and precise modulation of NSC behavior by neuronal inputs becomes possible. Alterations of neuronal signaling may induce temporary or persistent changes of the adult neurogenic process, such as increased neurogenesis by exercise (voluntary running) or reduced newborn neurons after social isolation [13]. The signal transmission from neurons primarily relies on the release of neurotransmitters or neuromodulators. Notably, neurotransmitters and neuromodulators are not separate molecular categories; rather, they represent functional classifications of molecules. For instance, dopamine acts as a neurotransmitter when controlling motor functions, while it serves as a neuromodulator when regulating synaptic dynamics. In the following, we will discuss the various neuron-derived signaling molecules.

### Glutamate promotes the activation of NSCs in SGZ

Glutamate is expressed by a majority of excitatory neurons in the brain. Hyperactivity of hippocampal neurons and aberrant glutamate dynamics have been reported in multiple neurodegenerative diseases. The persistent hyperactivities eventually exhaust NSC pools and reduce the long-term neurogenesis of affected brains [104]. Besides, although AD patients show defective neuronal activity at their late stage, clinical evidence indicates hippocampal neuronal hyperactivity in AD patients at earlier stages. Memory-associated brain scanning by functional magnetic resonance imaging of patients diagnosed with AD or mild cognitive impairment (MCI), revealed hippocampal hypoactivity in AD brains but hyperactivity in MCI brains, indicating that hippocampal hyperactivity precedes hypoactivity in AD. Such hyperactivity in AD is considered to be related to elevated glutamate levels caused by disturbed neuronal calcium signaling and reduced glutamate degradation by astrocytes, which potentially results in excessive glutamate within NSC niche [105]. In PD, neurodegeneration in the striatum disrupts the modulation of multiple output nuclei, resulting in hyperactivity of glutamatergic synapses [106]. Furthermore, glutamate neurotransmission is affected in HD and ALS [107, 108]. However, the correlation of neuronal hyperactivity with abnormal adult neurogenesis in these diseases remains to be fully understood.

Given the rapid turnover of glutamate in synaptic clefts, NSCs are proposed to be influenced by glutamate only when they are tightly surrounded by excitatory neurons. This is evidenced by the expression of glutamate receptors on SGZ NSCs but not on SVZ NSCs [103]. To explore the functions of the glutamate receptors on NSCs, kainic acid (KA), a glutamate receptor agonist, was injected into the dentate gyrus at a low dose, and a large increase of Nestin-expressing NSCs was observed. However, KA administration at a high dose that mimics severe epilepsy depletes the NSC pool rapidly and increases NSC-to-astrocyte differentiation [109]. In real situations, seizures prolonged for days can release a large amount of glutamate and other cytokines into the neural environment [110], which is considered to be associated with the significant expansion of active NSCs and neural progenitors in SGZ [109]. Additionally, Mossy cells, a unique glutamatergic neuron type in the dentate gyrus which is next to the SGZ, project tightly around the distal processes of quiescent NSCs. Excessive mossy cell activity induced by pharmacological activation increases the activation of quiescent NSCs [111]. Therefore, the excessive glutamate found in neurodegenerative diseases may act as a strong promoter of NSC consumption and contributes to NSC pool depletion (Fig. 1). Developing long-term therapies to decrease hyperactive neuronal excitability at early disease stages and trigger neurogenesis at late stages could be a promising direction for clinical research.

### Gamma-aminobutyric acid (GABA) transmission mediates the quiescence of adult NSCs

GABA is the principal inhibitory neurotransmitter in the developed brain, mainly expressed by inhibitory interneurons. Aberrant GABAergic transmission is recently regarded as a shared pathological feature across neurodegenerative diseases. Electrophysiological recordings of cell membranes from temporal cortices of AD patients revealed an age-dependent reduction of GABA currents, with the reductions being larger in membranes from younger patients, indicating an early onset of GABA disruption [112]. The deficiency of hippocampal GABA signals due to the hypoactivity of parvalbumin (PV)-positive interneurons, leads to hyperactivity of excitatory neurons, increasing the susceptibility to seizures in AD model at the early stage [113]. In fact, the deficiency of PV^+^ interneuron-derived GABA signals has been observed in multiple AD mouse models [114–116]. Similarly, postmortem samples from HD patients consistently showed a decline of GABA transmission [117]. HD animal research suggests that the decline results from reduced GAD67 expression [118]. Differently, in PD, both patients and mouse models display significant increases in GABA transmission and GAD65/67 expression [119]. Collectively, clinical observations reveal that GABAergic signaling is imbalanced in multiple neurodegenerative diseases. Effects of GABAergic signals on NSC activity and neurogenic processes have been reported.

Under physiological conditions, GABA acts as an essential inhibitory cue to constrain NSC activation to maintain long-term homeostasis. NSCs in both niches express GABA receptors to sense local inhibitory tones. In the SGZ, tonic GABA release from PV^+^ interneurons sustains NSCs in quiescence through GABA_A_ receptors. NSC-specific deletion of GABA receptor induces rapid activation of quiescent NSCs into systematic mitosis, while activation of PV^+^ interneurons is sufficient to restore the quiescent state of NSCs [120]. In the SVZ, GABA is secreted by neuroblasts after depolarization to inhibit NSC development and balance the neurogenic activity [103, 121]. Due to the decreased number or dysfunction of PV^+^ interneurons in AD animal models, the lack of enough GABA transmission in NSC niches could rapidly activate quiescent NSCs into massive proliferation at the early stage of AD as observed in previous studies. Notably, these overactivated NSCs tend to enter a glial cell fate instead of becoming neurons, merely consuming and exhausting the NSC pool of AD models [114–116]. To summarize, GABAergic signaling serves as a key homeostatic brake on adult neurogenesis that links neuronal activities to neurogenic processes.

### Monoamine neuromodulators that regulate adult neurogenesis

Neurons expressing monoamine neurotransmitters, including serotonin, dopamine and noradrenaline, are distributed relatively far from NSC niches. However, some of them can also mediate the quiescence and activation of NSCs via long-range projections from various brain regions. In neurodegenerative diseases, dysregulation of these neurotransmitters within NSC niches may also mediate abnormal neurogenic activities. Serotonin receptors are expressed by NSCs in both niches. Activation of serotonin receptors induces NSC proliferation and promotes generation of neurons. Impaired serotonin activities have been observed in AD, PD and epilepsy [122–125]. Dopaminergic neurons located in the midbrain can project to SVZ and develop synaptic connections to NSCs and other precursor cells. Stimulation of dopamine receptors seems to activate the NSC pool and induce cell expansion, but the accurate relationship is still under debate because the 14-day administration of dopamine D2 receptor antagonist also increased the number of proliferating NSCs [126, 127]. Dopamine deficiency, which is the key symptom of PD, also causes hypoactivity of PV^+^ interneurons in AD models. Clinical studies further illustrated reduced dopamine expression in the brains of MS patients [128–130]. In terms of circuit regulation, SGZ receives noradrenergic projections that promote the expansion of quiescent NSC pool [103]. Loss of noradrenergic projections has been widely detected in various neuropathies like AD, PD, ALS and MS, suggesting its link to neurodegeneration [131–134]. Overall, as NSCs express a wide range of neurotransmitter receptors, they can respond to any subtle alteration of neuronal activity within the microenvironment, which makes them susceptible to early pathological changes.

## Non-cellular components underlying NSC dysregulation

Apart from cellular components within NSC niches, non-cellular parts, especially the ECM and the oxygen environment, can also potentially modulate NSC activities.

### ECM induces neurogenic disruptions in neurodegenerative diseases

The ECM is composed of various biomacromolecules, providing mechanical support between cells and serving as a critical structural component of the brain. Alterations in the ECM composition lead to changes in its mechanical properties (e.g., stiffness, elasticity) and affinity for signaling molecules, thereby influencing the biochemical composition of the NSC microenvironment. Deficits of ECM have been widely reported in neurodegenerative diseases. Perineuronal nets (PNNs), an ECM component that is often associated with a subset of PV^+^ interneurons, are predominantly distributed in the cortex, substantia nigra and hippocampus [135, 136]. A PNN component called brevican is upregulated in the hippocampus of AD patients, which makes the PNN absorb more Aβ plaques and inhibits long-term potentiation [137]. PNN-encapsulated PV^+^ interneurons specifically show age-dependent reductions of GABA_A_ receptor expression in AD mice, which is sufficient to induce AD-associated anxiety [138]. Generally, PNN binds with α-synuclein and supports its degradation. However, deconstruction of PNN found in PD patients leads to α-synuclein accumulation in the extracellular space [139]. It is also reported that the expression patterns of ECM-composing macromolecules including PNN are altered in epilepsy (hippocampal sclerosis) or MS [139, 140].

Mechanical properties of the surrounding environment like stiffness, viscoelasticity or topology, are always a crucial regulator of stem cells. NSCs cultured on stiff material show overexpression of YAP (Yes-associated protein), which binds to β-catenin and represses the neurogenic process [141]. In particular, ECM with high stiffness imposes strong mechanical stress on NSCs and upregulates the expression of genes related to membrane cytoskeletal formation within NSCs, including *Sptbn1* encoding the protein β Spectrin II. The β Spectrin II activates the expression of EGR1 (early growth response protein 1) in NSCs, which suppresses neurogenesis but supports astrogliosis [142]. In addition, ECM indirectly regulates neurogenesis through its unique affinity to different chemicals. In SVZ, the ECM captures and absorbs growth factors, including FGF2 (fibroblast growth factor 2) and BMP4/7, via fractone-associated heparan sulfate binding, blocking their regulatory effects on NSCs through physical isolation. Consistently, a study on autism showed that reduced heparan sulfate is correlated with high expression of proliferative marker Ki67 in postmortem brain tissues from autism patients [143, 144]. However, straightforward evidence of ECM regulation of NSCs in the hippocampus is largely lacking, while research on neuroblastoma, a brain cancer generated from NSCs, noticed ECM regulation of the proliferation and differentiation of oncogenic NSCs [145]. The stiffness of ECM may affect NSC states via mechanical pressure, but this remains to be fully elucidated. Furthermore, despite detailed reports on ECM alterations in various neurodegenerative diseases, the mechanisms through which these changes affect the activity of NSCs and contribute to the progression of neurodegeneration require further research.

### Oxygen homeostasis modulates the NSC state

General hypoxia is commonly reported in brains of patients with neurodegenerative diseases. Notably, the mechanisms underlying hypoxia largely vary across distinct diseases. As one frequent complication of AD, sleep disruption creates a mild hypoxic brain environment, which leads to defective neuronal activity, chronic neuroinflammation and excessive oxidative stress [146]. Both the frequent occurrence of dyspnea and the elevation of hypoxic signals (e.g. HIF2α) in PD patients suggest a chronic hypoxic body environment [147–149]. Intracellular mitochondrial dysfunction is a critical contributor to hypoxia in HD [150]. ALS patients have lower plasma oxygen levels due to reduced functions of respiratory muscles, while the dysfunctional cerebrovascular system of MS patients causes hypoxia around demyelinated regions [151, 152]. The blood oxygen saturation is much lower in epilepsy patients due to sleep apnea, compared with that in the healthy population [153].

Interestingly, extracellular oxygen supply is identified as an important regulator of neurogenesis. Quiescent NSCs predominantly rely on glycolysis for energy supply, while fatty acid oxidation can also provide some energy. Once NSCs are activated, the activity of mitochondrial electron transport chain drastically rises to meet the high energy demand of cell mitosis and differentiation [13]. As a result, environmental oxygen accessibility regulates NSC activity dose-dependently. Mild hypoxia (2.5%–5% O_2_) is observed as an enhancer of quiescent NSC self-renewal and maintenance, while a severe hypoxic environment (1% O_2_) greatly blocks the mitosis of NSCs and even induces apoptosis [154]. Moderately elevated reactive oxidative species (ROS) could drive the proliferation of NSCs and increase the number of clonal neurospheres by activating the PI3K/Akt pathway [155]. In contrast, high environmental oxygen level (20% O_2_) significantly blocks the expression of HIF-1α and activated p53 in NSC, which lead to NSC pool depletion like the excessive oxygen supplementation does after hypoxic-ischemic brain injury [156, 157]. A transplantation-based study indicates that exposing cultured NSCs to physiologically low oxygen tension (3% O_2_) before surgery significantly elevates the proliferation and survival of transplanted NSCs [158].

In summary, a proper oxygen tension is essential for normal NSC functions. Both extremely low and high environmental oxygen levels are detrimental to maintaining long-term adult neurogenesis. However, despite the direct or indirect evidence that brain hypoxia in neurodegenerative diseases may support NSC activation and proliferation, the absolute ranges of oxygen tension in different brain regions remain unclear. Therefore, how changed oxygen accessibility in different diseases contributes to aberrant neurogenesis is still a large question mark.

## Clinical implications of deciphering adult NSC microenvironments

Neuronal impairment is the core manifestation of neurodegenerative diseases. Damage to neural circuits disrupts essential brain functions and triggers chronic inflammation that further accelerates neuronal loss. Considerable efforts have been directed toward complementary regenerative strategies. Although a study implies that direct restoration of endogenous neurogenesis in animal models of neurodegenerative diseases could improve symptoms in mice [116], translational research in humans remains highly restricted. Considering that most human patients are diagnosed at their advanced stages of neurodegenerative diseases, their NSC niches may have already been compromised. This not only limits the expected efficacy of activating endogenous neurogenesis, but is also associated with a potential risk of further depleting the NSC pool. Transplantation approaches are therefore a promising translational extension.

Currently, potential human pluripotent stem cell (hPSC)-derived products include embryonic stem cells (ESCs), mesenchymal stem cells, induced pluripotent stem cells (iPCSs) and NSCs. Notably, progenitor cells used in early transplantation studies were obtained from embryonic or neonatal rodent brains rather than from adult niches [159]. In a recent study, hPSCs were directed toward A10-like midbrain dopaminergic neurons through complicated growth-factor induction. Transplantation of these neurons into aged or diseased brains replenished damaged neurons, partially restored neural circuitry and showed anxiolytic and antidepressant-like phenotypes in transplanted mice [160]. In recent years, transplantation of hPSC-derived NSC organoids has displayed better therapeutic effects than 2D layers of cultured cells in rodent models, raising the attention to clinical trials of stem cell therapies [161].

However, according to current human trial reports, transplantation of hPSCs or specified neuronal progenitors failed to produce significant improvements in cognitive function or pathological biomarkers, although no adverse effects were reported in patients [159, 162]. More recently, while several stem cell-based products have proceeded into human testing, current results remain modest and often show only safety or biomarker changes rather than robust symptomatic improvement on patient life quality [9–12]. Several reasons have been proposed, including cell type mismatch with clinical indications, a lack of glial cell supplement and the deficiency of new neuronal connection [161]. The attempts to merely transplant and complement neural precursors are not sufficient to arouse the full potential of stem cell-based therapies.

Here, we emphasize that pathological alterations in the adult NSC microenvironment may represent a major factor limiting the efficacy of stem cell therapies for neurodegenerative diseases. Although hPSC or neuronal progenitor transplantation is usually supported by culture media supplemented with defined growth factors to sustain proliferation and differentiation, these factors are rapidly diluted or metabolized within the living tissue, making it difficult to persistently reconstruct a functional NSC niche. In such conditions, even successfully engrafted hPSCs or neuronal progenitors may undergo cell cycle arrest or premature depletion under the influence of a pathogenic environment that promotes activation, supports astrogliosis or is filled with chronic inflammatory storm, ultimately losing their regenerative potential.

In contrast, preclinical animal studies of stem cell therapies often show more pronounced efficacy [159–161], potentially due to the shorter timeframe for data collection and the relatively larger graft volumes that transiently overcome adverse microenvironmental cues. Under these well-controlled conditions, the detrimental impact of a diseased NSC niche is minimized, allowing for better xenograft survival and more apparent therapeutic benefits. To improve clinical outcomes, developments on therapeutic strategies should thus focus on not only functional cell replacement but also reconstructing or modulating the host microenvironment. Approaches such as delivering microenvironmental modulators (e.g., anti-inflammatory cytokines, Wnt/BMP regulators, or GABAergic enhancers), engineering ECM-mimetic scaffolds, or co-transplanting supportive glial cells could help stabilize the transplanted niche. Clinical attempts such as transplanting stem cells within artificial ECM scaffolds pretreated with proper neurotrophins, cytokines or even supportive glial cells may achieve more promising therapeutic efficacy [163]. Furthermore, targeting the pathological microenvironment may also protect the remaining endogenous NSC pool, thereby enhancing the self-repair capacity and slowing disease progression.

## Perspectives on NSC research in neurodegenerative diseases

Adult neurogenesis is now recognized as a fundamental component of adult brain plasticity, driven by NSCs that reside in the SGZ and SVZ and remain largely quiescent to preserve their long-term regenerative capacity. The activation, proliferation, and differentiation of NSCs rely on a precisely coordinated microenvironment where cellular regulators, neurotransmitters, ECM properties, and metabolic cues jointly determine the persistent equilibrium between quiescence and activation. This niche acts as a dynamic microecosystem capable of integrating biochemical, electrophysiological, and mechanical signals to maintain life-long neurogenesis.

In multiple neurodegenerative diseases, this delicate equilibrium collapses. Accumulating evidence indicates that adult neurogenesis is remodeled across major neurodegenerative diseases, although the primary pathogenic regions vary by pathology (Table 1). In AD, quantitative post-mortem studies consistently reported a marked decline of immature neurons in the dentate gyrus, highly correlating with progressive cognitive impairment [8, 39]. Ealy attempts to transplant proliferative NSCs into 3 × Tg AD mice partially rescued the cognitive behaviors [164]. In conditions characterized by motor dysfunction such as PD, HD and ALS, neural alterations extend to cognition-related brain regions as the patients show impaired brain functions at the late stage of disease [164–166]. SVZ/SGZ neurogenesis is reduced in PD, while stem cell-based therapies are capable of improving cognitive performance in animal models [6, 160]. SVZ neurogenic activity is increased in HD, which appears compensatory yet insufficient for endogenous striatal repair [167]. Transplantation of hESC-derived GABA medium spiny neurons in the striatum corrected motor deficits of HD model mice [168]. Neurogenic niches are also perturbed in ALS, with increased SVZ NSC proliferation and reduced SGZ neurogenesis associated with TDP-43 pathology [169]. Nevertheless, since the conventional ALS lesion region is spinal cord, current stem cell-based therapies transplant progenitors to the spinal cords of model animals instead of SVZ/SGZ; but notably, the progenitor-derived neurotrophic factors are also potential to affect the neurogenic process [170].

**Table 1:** Summary of NSC regulators and their alterations in neurodegenerative diseases

Signaling	Effect	Alteration	Reference
Notch1	Maintain quiescence	Decrease in AD	Marathe et al. [24]
		Decrease in PD	Desplats et al. [27]
		Increase in ALS	Nonneman et al. [26]
		Increase in PD	Dong et al. [28]
		Mutation in NIID	Shi et al*.* [29]
		Mutation in PD	Sone et al*.* [30]
		Mutation in CADASIL	Ehret et al*.* [31]
Wnt	Promote activation	Increase in AD	Bai et al*.* [43]
		Decrease in PD	Rmakrishna et al. [44]
		Decrease in HD	
		Decrease in ALS	
BMP	Maintain quiescence	Decrease in AD	Bai et al. [43]
		Increase in HD	Akbergenova and Littleton [47]
		Increase in ALS	Russo and Wharton [48]
EGF	Promote activation	Increase in AD	Choi et al. [64]
		Decrease in PD	O'Keeffe et al. [57]
		Decrease in HD	Marottoli et al. [58]
		Decrease in ALS	Younes et al. [59]
BDNF	Promote activation	Decrease in AD	Phillips et al. [67]
		Decrease in PD	Khalil et al. [68]
		Decrease in HD	Müller [69]
PTN	Promote activation	Decrease in AD	Nikolakopoulou et al. [71]
		Decrease in PD	
		Decrease in HD	
		Decrease in ALS	
		Decrease in HIV-induced neurocognitive disorders	
Inflammatory cytokine	Uncertiain	Increase in AD	Swardfager et al. [91]
		Increase in PD	Mogi et al. [93]
		Increase in ALS	Michaelson et al. [101]
		Increase in MS	Centonze et al. [102]
Glutamate	Promote activation	Increase in Epilepsy	Jessberger and Parent [104]
		Increase in early AD; Decrease in late AD	Targa Dias Anastacio, Matosin and Ooi [105]
		Increase in PD	Campanelli et al. [106]
		Increase in HD	André et al. [107]
		Increase in ALS	Xie et al. [108]
GABA	Maintain quiescence	Decrease in AD	Li et al. [114]; Verret et al. [115]
		Decrease in Epilepsy	Mao et al. [113]
		Decrease in HD	Hsu, Chang and Chern [171]
		Increase in PD	Muñoz, de la Fuente and Sánchez-Capelo [119]
Serotonin	Promite activation	Decrese in AD	Wu et al. [123]
		Decrease in PD	Pagano et al. [124]
		Decrease in Epilepsy	Pottoo et al. [125]
Dopamine	Uncertain	Decrease in PD	Lees, Hardy and Revesz [128]
		Decrease in AD	Spoleti et al. [129]
		Decrease in MS	Carotenuto et al. [130]
Noradrenaline	Promote activation	Decrease in AD	Gutiérrez et al. [131]
		Decrease in PD	Sommerauer et al. [132]
		Decrease in ALS	Scekic-Zahirovic et al. [133]
		Decrease in MS	Polak, Kalinin and Feinstein. [134]
PNN	Uncertain	Increase in AD	Howell et al. [137]
		Decrease in PD	Dong et al. [139]
		Decrease in MS	
		Decrease in Epilepsy	Sitaš et al. [140]
Oxygen tension	Promote activation	Decrease in AD	Liu et al*.* [146]
		Decrease in PD	Burtscher et al. [172]
		Decrease in HD	Burtscher et al. [150]
		Decrease in ALS	Swindell et al. [151]
		Decrease in MS	Halder and Milner [152]
		Decrease in Epilepsy	Carosella et al. [153]

Clinical and preclinical observations support the notion that neurogenic dysregulation is a widespread but heterogeneous response to neurodegeneration, potentially contributing indirectly to circuit dysfunction in diseases where pathology lies outside SGZ or SVZ. However, although stem cell-based interventions have shown encouraging outcomes in animal models, clinical results remain disappointing. The inability of transplanted hPSCs or NSC-derived progenitors to restore cognitive function suggests that the disruptions of cytokines, local neurotransmission and other non-cellular components compromise the survival and integration of transplants.

**Fig. 2:**
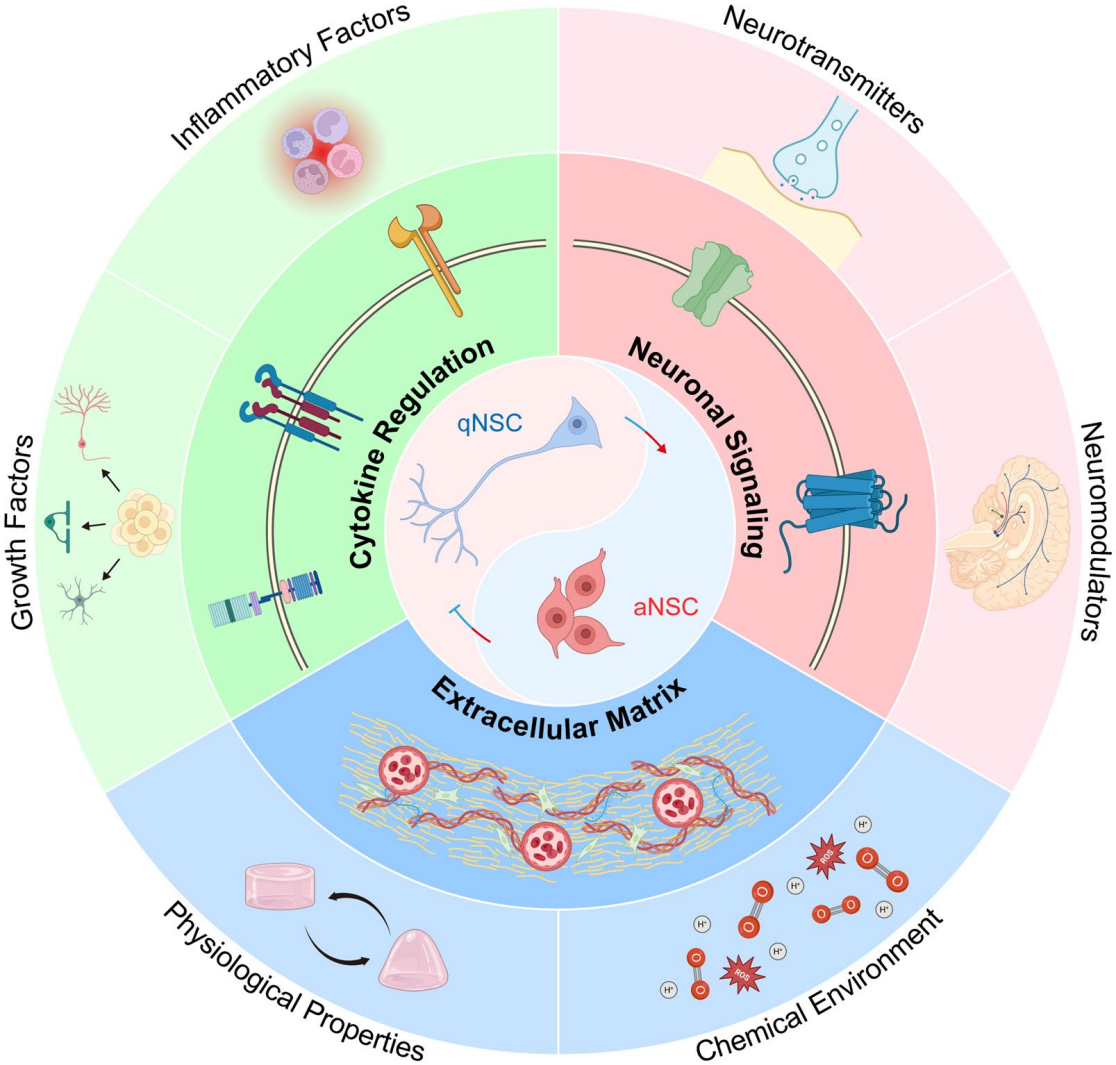
Diagram of disrupted environmental NSC regulators in diseases. The center Yin and Yang fish represents the balanced transition between quiescent and activated NSCs, surrounded by niche regulators within the NSC microenvironment. Disease-associated niche regulators in the NSC niche are categorized into 3 dimensions according to their modes of function, which are further divided into 2 subgroups, respectively

Current clinical evidence indicates multiple disease-associated environmental regulators of neurogenesis, including Notch-, BMP/Wnt-, EGF-, and neurotransmitter-dependent pathways, while chronic inflammation, neuronal excitatory–inhibitory imbalance, ECM remodeling, and hypoxic stress also reshape the microenvironment of NSCs. These pathological conditions accelerate NSC depletion and impair the generation and integration of newborn neurons, raising the possibility that defective neurogenesis is not merely a downstream consequence but also a contributing factor to neurodegenerative progression (Fig. 2). However, our understanding of the unique microenvironment associated with each disease is still limited. High-throughput technologies such as single-cell and spatial transcriptomics, proteomics, and multi-omics integration, will facilitate systematic mapping of the molecular and cellular heterogeneity of NSC niches in physiological and pathological states, leading to biomarker discovery and therapeutic target identification. Given that the NSC microenvironment is widely disrupted in neurodegenerative diseases, future therapeutic strategies should prioritize reconstruction of the NSC microenvironment to resemble the physiological state.

## Conclusion

Neurogenesis is disrupted in neurodegenerative diseases. Alterations of NSC regulators have been reported in neurodegenerative diseases, but whether and how the disease-associated NSC regulators disrupt the neurogenic environment remain largely unknown. Clinical trials of stem cell transplantation in neurodegenerative diseases have shown modest effects. Therapies combining stem cell transplantation with long-term environmental remodeling through morphogen/growth factor modulation, microglial activation state rebalancing, restoration of neuronal excitatory–inhibitory balance, ECM engineering, enhancing metabolic support, or vascular stabilization may improve the efficacy of regenerative interventions. Restoring the microenvironments may also revive residual endogenous NSCs and promote self-repair. Ultimately, re-establishing a physiological NSC niche offers a compelling direction for future regenerative therapy.

## Data Availability

Not applicable.

## Abbreviations

AD: Alsheimer's disease
ALS: Amyotrophic lateral sclerosis
Aβ: Amyloid β
BDNF: Brain-derived neurotrophic factor
BMP: Bone morphogenetic protein 3/4/7
CADASIL: Cerebral autosomal dominant arteriopathy with subcortical infarcts and leukoencephalopathy
CSF: Cerebrospinal fluid
Cx3cr1: C-X3-C Motif Chemokine Receptor 1
ECM: Extracellular matrix
EGF: Epidermal growth factor
GABA: Gamma aminobutyric acid
HD: Huntington's disease
hPSC: Human pluripotent stem cell
IGF-1: Insulin-like growth factor 1
IPC: Intermediate progenitor cell
iPSC: Induced pluripotent stem cell
KA: Kainic acid
MCI: Mild cognitive impairment
MS: Multiple sclerosis
NIID: Neuronal intranuclear inclusion disease
NOTCH2NLC: Notch homolog 2 N-terminal-like protein C
NSC: Neural stem cell
PD: Parkinson's disease
PNN: Perineuronal net
PTN: Pleiotrophin
PV: Parvalbumin
sFRP3: Secreted frizzled-related protein 3
SGZ: Subgranular zone
SMAD: SMA (sma for small body size in *C. elegans*) and MAD (mothers against DPP in *Drosophila*) related protein
SMAD4: SMAD family member 4
SVZ: Subventricular zone
TGF-α/β: Transforming growth factor α/β
TNF-α: Tumor necrosis factor α
TSPO-PET: Translocator protein positron emission tomography
